# Backbone and side‑chain ^1^H, ^13^C and ^15^N resonance assignments and secondary structure determination of the rhizobial FixJ

**DOI:** 10.1007/s12104-025-10221-w

**Published:** 2025-02-01

**Authors:** Akio Horikawa, Rika Okubo, Naoki Hishikura, Riki Watanabe, Kaori Kurashima-Ito, Pooppadi Maxin Sayeesh, Kohsuke Inomata, Masaki Mishima, Hiroyasu Koteishi, Hitomi Sawai, Yoshitsugu Shiro, Teppei Ikeya, Yutaka Ito

**Affiliations:** 1 Department of Chemistry, Tokyo Metropolitan University, 1-1 Minami-Osawa, Hachioji, Japan; 2 Present Address: Laboratory for Dynamic Biomolecule Design, RIKEN Centre for Biosystems Dynamics Research, RIKEN, Yokohama, Japan; 3 Present Address: Core for Spin Life Sciences, Okazaki Collaborative Platform, National Institutes of Natural Sciences, Okazaki, Japan; 4 Present Address: Department of Molecular Biophysics, School of Pharmacy, Tokyo University of Pharmacy and Life Sciences, Hachioji, Japan; 5 Graduate School of Life Science, University of Hyogo, Hyogo, Japan; 6 Present Address: Laboratory of Protein Crystallography, Institute for Protein Research, Osaka University, Osaka, Japan; 7 Division of Chemistry and Materials Science, Graduate School of Integrated Science and Technology, Nagasaki University, Nagasaki, Japan; 8 Institute for Molecular Science, National Institutes of Natural Sciences, Okazaki, Japan

**Keywords:** Two component regulatory system, Response regulator, Nitrogen fixation, Multi domain protein

## Abstract

The symbiotic nitrogen-fixing bacterium *Bradyrhizobium japonicum* (*B.japonicum*) enables high soybean yields with little or no nitrogen fertiliser. A two component regulatory system comprising FixL, a histidine kinase with O_2_-sensing activity, and FixJ, a response regulator, controls the expression of genes involved in nitrogen fixation, such as *fixK* and *nifA*. Only under anaerobic conditions, the monophosphate group is transferred from FixL to the N-terminal receiver domain of FixJ (FixJ_N_), which eventually promote the association of the C-terminal effector domain (FixJ_C_) to the promoter regions of the nitrogen-fixation-related genes. Structural biological analyses carried out so far for rhizobial FixJ molecules have proposed a solution structure for FixJ that differs from the crystal structures, in which the two domains are extended. To understand the FixJ activation caused by phosphorylation of the N-terminal domain, which presumably regulates through the interactions between FixJ_N_ and FixJ_C_, here we have performed backbone and sidechain resonance assignments of the unphosphorylated state of *B. japonicum* FixJ.

## Biological context

Rhizobia are bacteria that can live in the roots of leguminous plants and form rhizoids. Rhizobia have a symbiotic relationship in which they convert atmospheric nitrogen molecules into ammonia (nitrogen fixation) by nitrogenases, which is supplied to plants, and receive photosynthetic products from the plants. Since nitrogenase is quickly inactivated when exposed to molecular oxygen, rhizobium bacteria have a sophisticated two component regulatory system (TCS) (Urao et al. 2000; Chauhan and Calderone 2008; Gotoh et al. 2010) consisting of two proteins, FixL and FixJ (Gilles-Gonzalez et al. 1991; Gong et al. 1998), to express genes involved in nitrogen fixation only in the absence of O_2_ (David et al. 1988).

*Bradyrhizobium japonicum* forms symbiotic relationships particularly with soybeans. *B. japonicum* FixL is a histidine kinase (HK) comprising N-terminal tandem Per-Arnt-Sim (PAS) domains, PAS-A and PAS-B, followed by a C-terminal dimerisation and histidine phosphotransfer (DHp) domain and a catalytic adenosine triphosphate-binding (CA) domain. Unlike many other TCS HKs, which are integrated into the membrane, *B. japonicum* FixL is water soluble and cytoplasmic, and uses a haem *b* cofactor bound to the PAS-B domain to sense O_2_ (Gilles-Gonzalez et al. 1994; Gong et al. 1998). *B. japonicum* FixJ is a response regulator (RR) consisting of an N-terminal receiver (REC) domain (FixJ_N_) and a C-terminal effector domain (FixJ_C_) that binds to DNA.

Under anaerobic conditions the hydrolysis of ATP at the CA domain and the autophosphorylation at His-291 in the DHp domain occur. The monophosphate group is subsequently transferred to the sidechain of Asp-55 in FixJ_N_ through the FixL-FixJ interaction, which eventually promotes the association of the helix-turn-helix motif of FixJ_C_ to the promoter regions of nitrogen-fixation-related genes, such as *fixK* and *nifA* (Wright et al. 2018).

Structural biological analyses have been carried out for FixJ, including for related species. First, the structure of FixJ_N_ of *Sinorhizobium meliloti* was determined by X-ray crystallography (Birck et al. 1999; Gouet et al. 1999). Next, the solution structure of *S.meliloti* FixJ_C_ was determined by NMR, and its interaction with dsDNA containing fixK promoter sequence was also analysed (Kurashima-Ito et al. 2005). Recently the crystal structures of full-length FixJ of *B. japonicum* were determined, in which multiple interdomain orientations were found, caused by the bending of the α-helix connecting FixJ_N_ and FixJ_C_ at different angles around Ala-123 (Wright et al. 2018). However, it turned out that no crystallographic conformer fitted the experimental SAXS data for full-length FixJ well, which suggests that FixJ in solution has more compact interdomain orientations on average (Wright et al. 2018).

To understand the mechanism of FixJ activation caused by phosphorylation of the N-terminal domain, the interactions between FixJ_N_ and FixJ_C_ in solution need to be analysed in detail. Therefore, as a first step, we initiated the heteronuclear multi-dimensional NMR study of the unphosphorylated state of *B. japonicum* FixJ (BjFixJ) for the assignment of the backbone and sidechain NMR resonances, which will provide a framework for the further analysis of 3D structure and dynamics of BjFixJ in solution.

## Methods and experiments

### Protein expression and purification

The gene encoding *B. japonicum* FixJ (BjFixJ) was cloned into a pET-22b(+) vector (Novagen) as an N-terminal His-tag-SUMO-fusion protein, and the resulting plasmid was introduced into the *Escherichia coli* BL21 (DE3) star strain (Invitrogen). For NMR experiments, uniformly ^13^C and ^15^N-labelled (^13^C/^15^N-) and uniformly ^2^H, ^13^C and ^15^N-labelled (^2^H/^13^C/^15^N-) samples were prepared. For ^13^C/^15^N-labelling, the transformed bacteria were grown at 37 °C in M9 minimal medium containing 2 g/L [^13^C_6_] D-glucose (Cambridge Isotope Laboratories, Inc.) and 1 g/L ^15^NH_4_Cl (Cambridge Isotope Laboratories, Inc.) as the sole carbon and nitrogen sources, respectively. For ^2^H/^13^C/^15^N-labelling, the ^13^C/^15^N-labelled M9 medium was prepared with 95% D_2_O (Sigma-Aldrich). At a turbidity of ~ 0.5 OD (600 nm), expression of the protein was induced by the addition of isopropyl thio-β-D-thiogalactoside to a final concentration of 1 mM. After 18–20 h of further growth at 18 °C, the cells were harvested, washed and stored at -80 °C.

All the purification procedures described below were performed at 4 °C. The cells were washed once, centrifuged and resuspended to 0.1 g of cell paste/mL with lysis buffer [50 mM Tris-HCl (pH 8.0), 300 mM NaCl, 0.01% NP-40, 25% sucrose and protease inhibiter cocktail (Thermo Fisher Scientific)]. The cells were lysed with lysozyme (0.1 mg/mL) for 15 min, and the solution was incubated with DNase I (0.1 mg/mL), 0.5% NP-40 and 5 mM MgCl_2_ for 15 min. Further cell lysis was performed by using ultrasonication. The soluble and insoluble fractions were separated by centrifugation at 30000*g* for 30 min.

The His-tag-SUMO-BjFixJ was purified using a Ni–NTA agarose affinity column (QIAGEN) pre-equilibrated with purification buffer A [50 mM Tris-HCl (pH 8.0), 300 mM NaCl and 1 mM Tris (2-carboxyethyl)-phosphine (TCEP), 10% Glycerol]. The fractions eluted using a 0−250 mM imidazole (pH 8.0) gradient were collected, and the N-terminal His-tag-SUMO part was cleaved with an incubation for overnight with SENP2 protease which is prepared as a GST-fusion protein. The cleaved BjFixJ were obtained by collecting flow-through fractions from sequential Ni-NTA and Glutathione Sepharose (Cytiva) column chromatography steps. Further purification of BjFixJ was achieved using a Superdex 75 pg HiLoad™ 16/600 (Cytiva) gel-filtration column pre-equilibrated with purification buffer B [50 mM Tris-HCl (pH 8.0), 300 mM NaCl and 1 mM TCEP].

The purified protein samples were concentrated (0.3 mM) and dissolved in NMR buffer [50 mM Tris-HCl (pH 8.0), 300 mM NaCl] containing 10% D_2_O for NMR lock using Amicon Ultra-15 centrifugal filter units (Millipore). This buffer condition and the sample concentration were selected after optimisation, since BjFixJ showed concentration-dependent nonspecific association.

### NMR spectroscopy

All NMR experiments were performed at a probe temperature of 25 °C on a Bruker Avance-III HD 600 MHz spectrometer equipped with a cryogenic triple-resonance probehead. All 2D NMR spectra were processed using the Azara 2.8 software suite (Wayne Boucher and Department of Biochemistry, The University of Cambridge). For all 3D/4D NMR data, a non-uniform sampling scheme was used for the indirectly observed dimensions to reduce experimental time. Either quantitative maximum entropy (QME) (Hamatsu et al. 2013) or compressive sensing with CambridgeCS software (Mark J. Bostock, Robert Tovey and Daniel Nietlispach, Department of Biochemistry, The University of Cambridge) (Bostock et al. 2012) were used for processing non-uniformly sampled indirect dimensions. All spectra were visualised and analysed using the CcpNmr Analysis 2.5.2 software (Vranken et al. 2005).

**Fig. 1:**
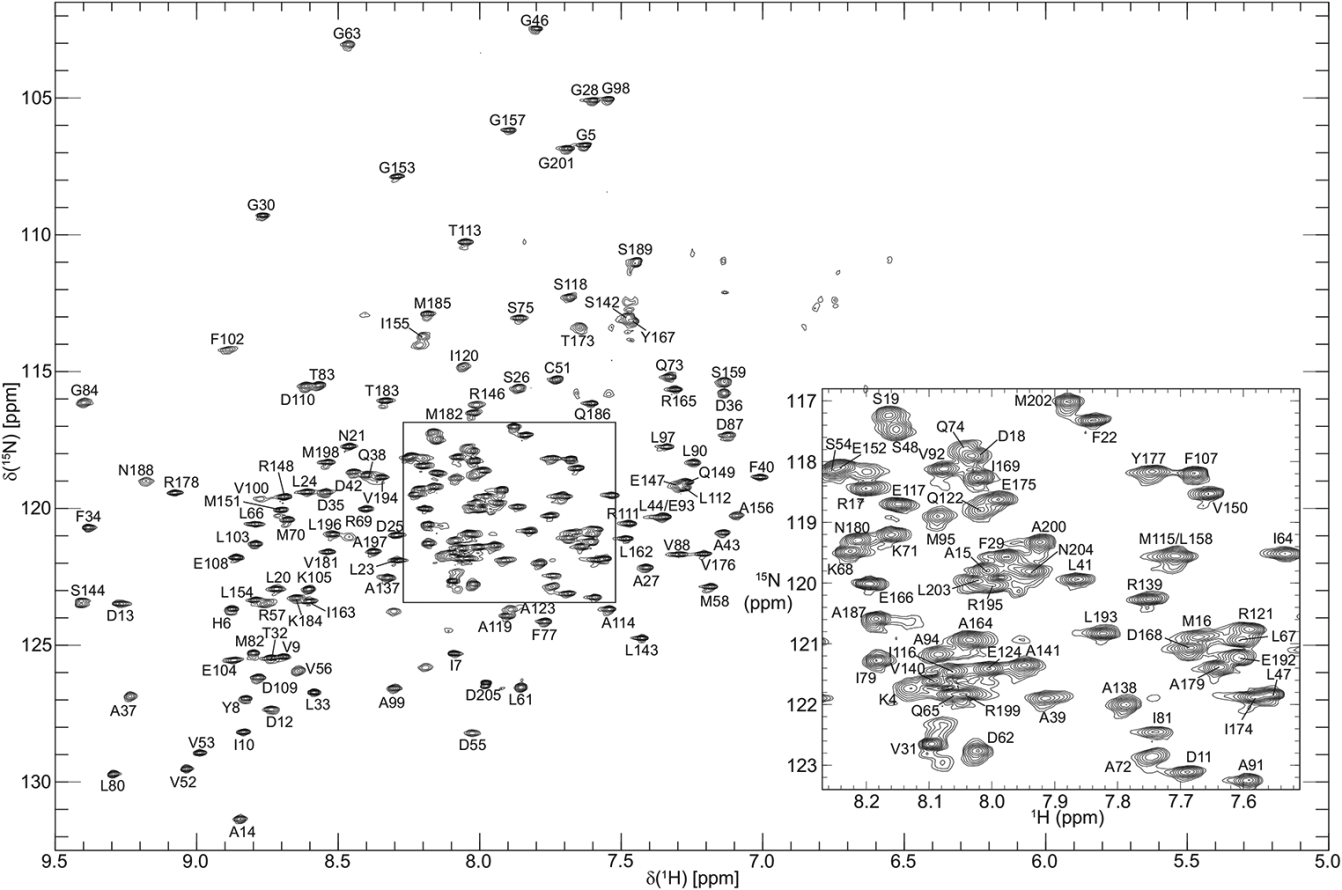
2D ^1^H-^15^N TROSY-HSQC spectrum of 0.3 mM ^2^H/^13^C/^15^N-labelled *B. japonicum* FixJ acquired on a Bruker Avance III HD 600 spectrometer at 25˚C, pH 8.0. Cross peaks are labelled with their corresponding backbone assignments

**Fig. 2:**
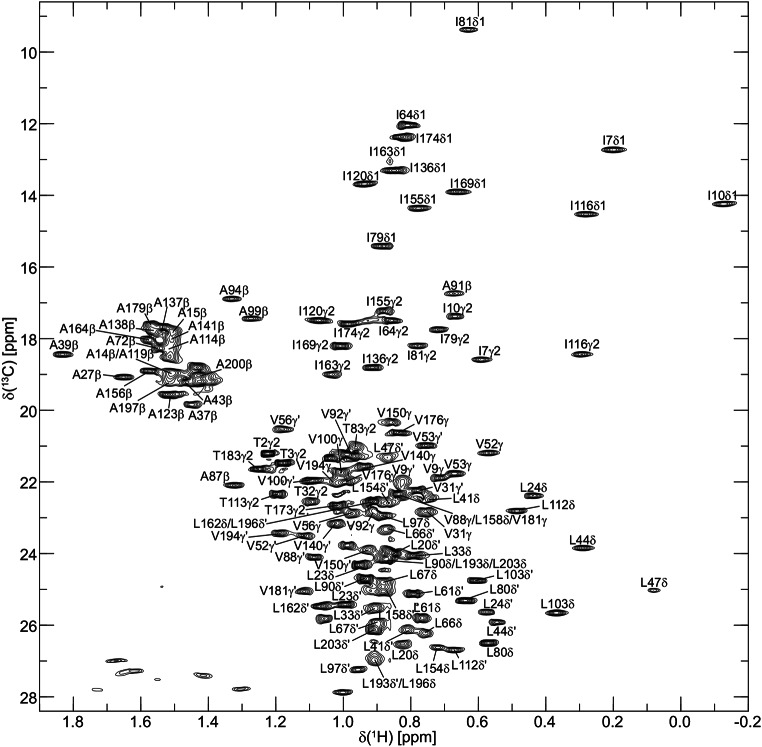
2D ^1^H-^13^ C constant-time HSQC spectrum of 0.3 mM ^13^C/^15^N-labelled *B. japonicum* FixJ acquired on a Bruker Avance III HD 600 spectrometer at 25˚C, pH 8.0. Mirror-image linear prediction in the *t*_1_ (^13^C) dimension was performed prior to Fourier transform. Only positive contours are shown. Cross peaks due to methyl groups of Ala, Ile, Leu, Thr, and Val residues are labelled with their corresponding assignments

Backbone ^1^H^N^, ^15^N, ^13^C^α^, ^13^C’, and sidechain ^13^C^β^ resonance assignments of BjFixJ were performed by analysing six 3D TROSY-type triple-resonance NMR spectra, HNCA, HN(CO)CA, HN(CA)CB, HN(COCA)CB, HN(CA)CO and HNCO measured on the ^2^H/^13^C/^15^N-labelled sample. The 2D ^1^H-^15^N-TROSY-HSQC spectrum of BjFixJ annotated with the assignments is shown in Fig. 1. Aliphatic sidechain ^1^H and ^13^C resonances were assigned by analysing 3D HBHA(CBCACO)NH, H(CCCO)NH, (H)CC(CO)NH, and HCCH-TOCSY experiments measured on the ^13^C/^15^N-labelled sample. As an example of the sidechain resonance assignment, the methyl-region of the 2D ^1^H-^13^C HSQC annotated with the assignments is shown in Fig. 2.

3D ^13^C-separated NOESY, 3D ^15^N-separated NOESY and 4D ^13^C/^15^N-separated NOESY experiments were used for assigning aromatic ^1^H and ^13^C resonances as well as further confirmation of the assigned sidechain chemical shifts.

### Extent of assignments and data deposition

^1^H, ^13^C, and ^15^N chemical shift assignments for BjFixJ were deposited in the Biological Magnetic Resonance Bank (BMRB) under the ID 26,351. As described, backbone resonance assignment was performed using the ^2^H/^13^C/^15^N-labelled sample, while sidechain resonances were analysed using the ^13^C/^15^N-labelled sample. For BMRB-deposition, chemical shifts for protonated ^13^C^α^ and ^13^C^β^ were used.

BjFixJ consists of 205 amino acid residues containing 9 proline residues. If there is no conformational multiplicity with a slow exchange regime, there are therefore a total of 195 observable ^1^H-^15^N correlation cross peaks due to backbone amide groups in the 2D ^1^H-^15^N TROSY-HSQC spectra, excluding the N-terminal amino group as well. For backbone resonances, we assigned them in the assignment completeness of 86.7% for ^1^H^N^ (169/195), 86.7% for ^15^N (169/195), 86.8% for ^13^C^α^ (178/205), 86.3% for ^13^C^β^ (177/205), and 87.3% for ^13^C’ (179/205).

**Fig. 3:**
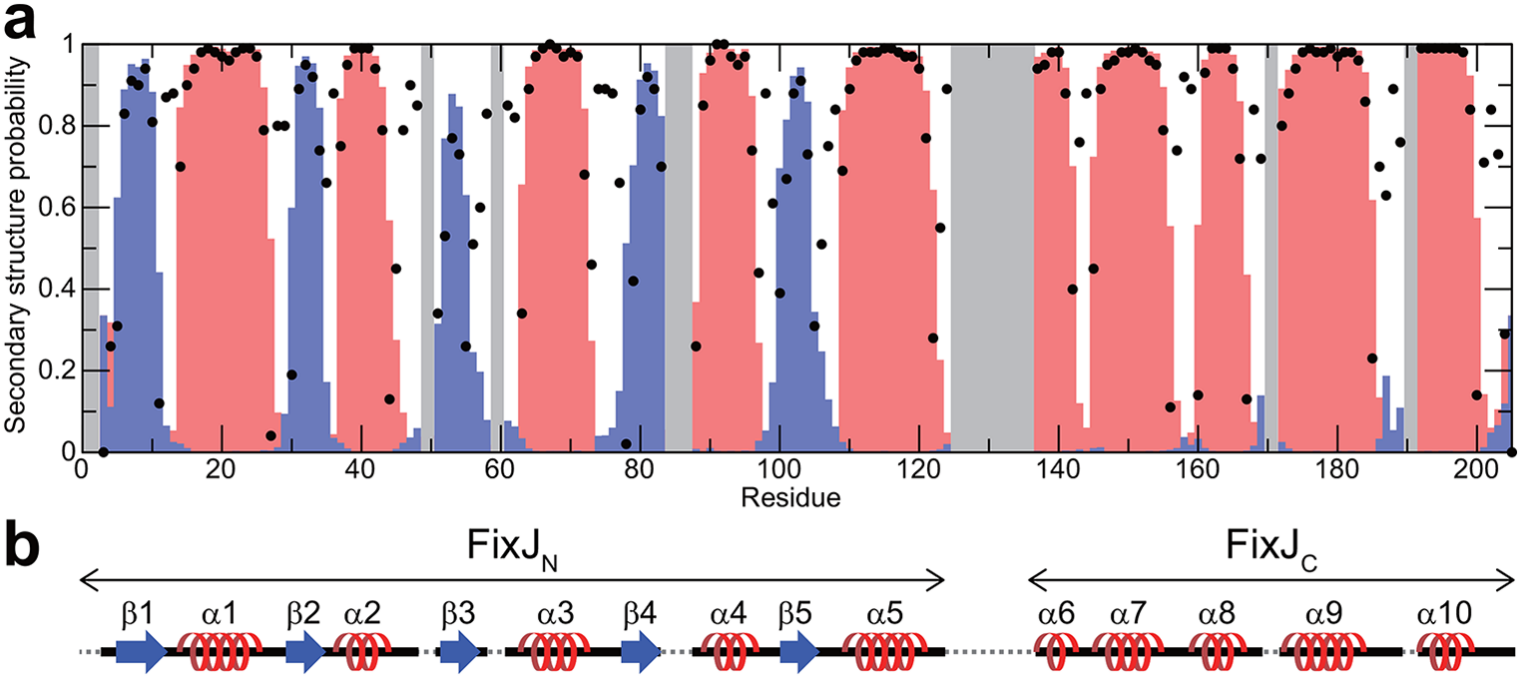
**a.** Secondary structure probabilities for *B. japonicum* FixJ predicted by TALOS-N (Shen and Bax 2013). Blue and red bars represent probabilities that the corresponding residues form β-strand or α-helix, respectively. The confidence in the prediction at each residue is indicated by a black dot. Grey regions represent the residues for which secondary structure prediction based on the chemical shifts was not performed due to the lack of assignments. **b**. Schematic diagram of the predicted secondary structures

The secondary structure of BjFixJ was predicted by TALOS-N (Shen and Bax 2013) based on the assigned resonances (Fig. 3). For FixJ_N_ and FixJ_C_ the βαβαβαβαβα and ααααα folds were found, which is consistent with previously reported structural biological results on FixJ as a whole and of each domain.

The incompleteness in the backbone resonance assignments was presumably caused by line broadening of cross peaks in the ^1^H-^15^N TROSY-HSQC spectrum, thus providing scarcely observable cross peaks in triple-resonance spectra for these ^1^H^N^-^15^N correlations. Incomplete exchange of amide ^2^H to ^1^H in the ^2^H/^13^C/^15^N-labelled sample was ruled out, since almost all the ^1^H-^15^N correlation cross peaks found in the ^1^H-^15^N HSQC spectrum of the protonated sample were indeed observed in the ^1^H-^15^ N TROSY-HSQC spectrum of the deuterated sample as well. The residues with missing amide-assignments were Thr-2, Thr-3, Phe-49, Gly-50, Gly-60, His-85, Gly-86, Lys-96, Asp-101, Ala-126-Ile-136, Asn-160, Lys-161, Ser-170, Arg-172, Leu-190 and Ser-191. These “unassigned” residues are all localised at the N-terminus (Thr-2 and Thr-3) or in loop regions between α2 and β3 (Phe-49 and Gly-50), β3 and α3 (Gly-60), β4 and α4 (His-85 and Gly-86), α4 and β5 (Lys-96 and Asp-101), α5 and α6 (Ala-126-Ile-136), α7 and α8 (Asn-160 and Lys-161), α8 and α9 (Ser-170 and Arg-172), and α9 and α10 (Leu-190 and Ser-191) and are therefore likely to be exposed to the solvent. Since the NMR experiments were carried out at pH 8, the exchange of amide protons due to these residues with the solvent presumably caused significant line broadening of these resonances. On the other hand, the Ala-126–Ile-136 residues were localised following Ala-123, which is the exact location where the bending of the α-helix connecting FixJ_N_ and FixJ_C_ was observed in the crystal structures. Thus changes in the relative orientation of two domains in solution may also explain the exchange broadening of the ^1^H^N^-^15^N correlation cross peaks for the residues. The predicted secondary structure, in which a loop structure was suggested for Ala-123 and Glu-124, also supports the flexibility of the region in solution with wider area than that observed in the crystal structures.

For sidechain ^1^H/^13^C resonances, roughly 84.5% of aliphatic and 54.5% of aromatic chemical shifts were identified. In addition 91.7% (11/12) of sidechain NH_2_ groups were assigned. The assignments obtained in this study will be used in the analysis of the detailed 3D structure and dynamics of the unphosphorylated FixJ. Comparison with NMR results of phosphorylated FixJ in the future should also help to understand the mechanism of FixJ activation caused by the Asp-55 phosphorylation.

## Data Availability

^1^ H, ^13^ C, and ^15^N chemical shift assignments for *Bradyrhizobium japonicum* FixJ were deposited in the Biological Magnetic Resonance Bank (BMRB) under ID 26351.
